# Identification of miR-34a-target interactions by a combined network based and experimental approach

**DOI:** 10.18632/oncotarget.9103

**Published:** 2016-04-29

**Authors:** Martin Hart, Stefanie Rheinheimer, Petra Leidinger, Christina Backes, Jennifer Menegatti, Tobias Fehlmann, Friedrich Grässer, Andreas Keller, Eckart Meese

**Affiliations:** ^1^ Institute of Human Genetics, Saarland University, 66421 Homburg, Germany; ^2^ Chair for Clinical Bioinformatics, Saarland University, 66123 Saarbrücken, Germany; ^3^ Institute of Virology, Saarland University Medical School, 66421 Homburg, Germany

**Keywords:** network analysis, PKC family, miR-34a, cancer, immune system

## Abstract

Circulating miRNAs have been associated with numerous human diseases. The lack of understanding the functional roles of blood-born miRNAs limits, however, largely their value as disease marker. In a systems biology analysis we identified miR-34a as strongly associated with pathogenesis. Genome-wide analysis of miRNAs in blood cell fractions highlighted miR-34a as most significantly up-regulated in CD3+ cells of lung cancer patients. By our *in silico* analysis members of the protein kinase C family (PKC) were indicated as miR-34a target genes. Using a luciferase assay, we confirmed binding of miR-34a-5p to target sequences within the 3′UTRs of five PKC family members. To verify the biological effect, we transfected HEK 293T and Jurkat cells with miR-34a-5p causing reduced endogenous protein levels of PKC isozymes. By combining bioinformatics approaches with experimental validation, we demonstrate that one of the most relevant disease associated miRNAs has the ability to control the expression of a gene family.

## INTRODUCTION

MicroRNAs (miRNAs) are small RNA molecules of around 20 nucleotides in length with enormous impact on cell function [1]. As of now, more than 28645 miRNA entries, including 2,588 human mature miRNAs are deposited in the miRNA Database (miRBase Release 21, http://www.mirbase.org/, [2]). Alterations in miRNA expression have been related to a yet increasing number of human diseases making these altered miRNAs both suitable starting points to better understand the biology of diseases and to develop disease indicating biomarkers [3]. Others and we recently reported miRNA signatures in blood of patients with various diseases including cancer, infertility, neurodegenerative and autoimmune diseases [4–8]. Many miRNA signatures show a high accuracy not only when comparing patients with healthy controls, but also for the comparison between patients with different diseases of the same organ like for example between lung cancer patients and patients with COPD [9].

Understanding the biological role of miRNAs that contribute to the diagnostic signature will significantly enhance the value of biomarker miRNA signatures. For serum or plasma based miRNA biomarkers it is impossible to determine the cells of origin that give rise to a miRNA signature. By contrast miRNA signatures that are derived from whole blood can potentially be attributed to a specific cell type or to a vesicle structure. This in turn can help to understand the cellular function of miRNAs that contribute to a miRNA pattern of diagnostic value. There have been various attempts to define miRNA expression patterns in human blood cells. Cell type specific miRNA expression patterns have been identified in nine different immune cell subsets [10]. The expression analysis of selected miRNAs allowed a clustering between reticulocytes, platelets, granulocytes, monocytes, B-cells, and T-cells [11]. Recently, we reported specific miRNA expression pattern for eosinophilic and neutrophilic granulocytes (CD15+), monocytes (CD14+), B-cells (CD19+), T-cells (CD3+), and natural killer (NK) cells (CD56+) both in healthy individuals and in lung cancer patients [12].

Here we analyzed hsa-miR-34a that showed a significantly increased abundance in the CD3+ T-cells of lung cancer patients as compared to healthy individuals. There is ample evidence that miR-34a is a tumor suppressor with lost or reduced expression levels in many cancers [13]. MiR-34a has also been suggested as an ideal therapeutic means against tumor metastasis and recurrence. Despite its numerous cellular effects as for example on apoptosis, the exact downstream mechanisms of miR-34a are largely not understood. There is an extended number of target genes, which are, however, mostly predicted and await experimental confirmation. Without such confirmation, reported biological implication can eventually be due to off target effects as a miRNA can bind multiple target genes and thus reduce their expression. We specifically analyzed members of the protein kinase C (PKC) family as possible targets of miR-34a. Unquestionably, PKCs play a crucial role, which can hardly be underestimated - even as compared to other kinases- both for physiological and pathological processes including cancer as shown by a multitude of studies [14–20]. The understanding of their specific impact on cellular processes is, however, complicated by the various PKC isozymes, which show multifaceted (inter)actions. By analyzing the regulatory effects of miR-34a on PKC members, we contribute to the understanding of the regulation of this highly important kinase family [21–26].

## RESULTS

### Network analysis & target prediction

**Figure 1:**
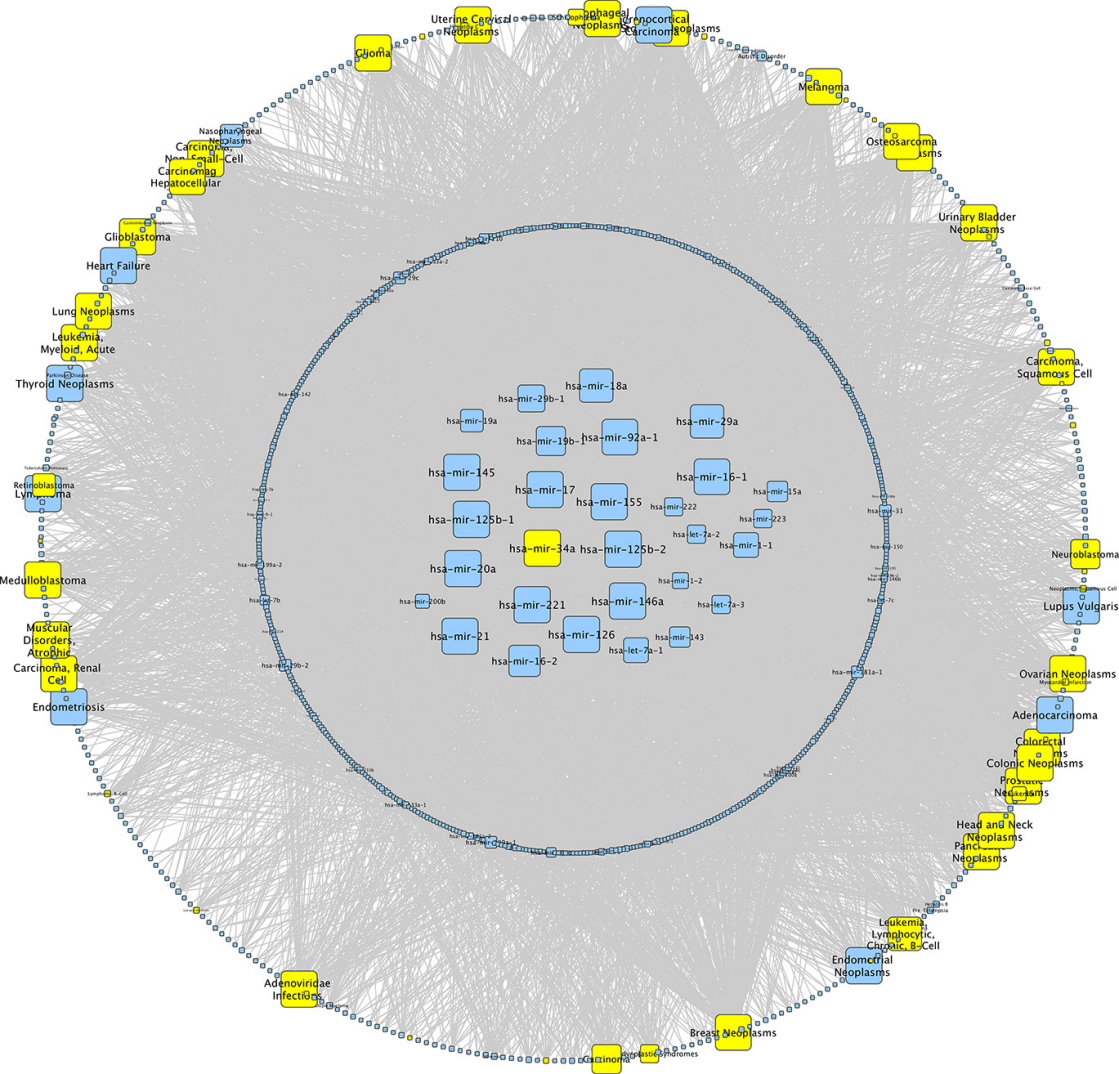
Network analysis of miR-34a We performed a network analysis for miR-34a over all datasets of the Human microRNA Disease Database (HMDD v2.0). The size of the knots mirrors the number of connections whereat the maximal size of the knots correspond to > 50 connections. MiRNAs in the center are associated with > 40 different diseases and miRNAs in the circle around the center with < 40. The outer circle displays all diseases whereat diseases associated with miR-34a are highlighted in yellow.

To discover miRNAs that are most important as disease biomarkers, we performed a network analysis over all datasets of the Human microRNA Disease Database (HMDD v2.0) [27]. We identified miR-34a as one of the miRNAs that are most frequently associated with human diseases (Figure 1). For the identification of the specific blood component significantly contributing to altered miRNA abundance, we reinvestigated the expression of miRNAs in different leukocyte subpopulations, which were taken from healthy controls and from lung cancer patients [12]. In CD3+ T-cells we found 13 miRNAs that showed a significantly increased abundance in lung cancer patients as compared to healthy individuals. Out of these miRNAs we found 3 miRNAs with a fold change larger than 1.5 and the highest fold change was observed for hsa-miR-34a (Table 1). We performed an *in silico* analysis to predict target genes for miR-34a using miRWalk 2.0 [28]. For further analysis we used only genes which were predicted by at least 4 algorithms. Thereby we identified 3179 genes including PKC family members as potential targets for miR-34a.

**Table 1:** Upregulated miRNAs in CD3+ cells of LCa patients vs. healthy donors

Upregulated miRNAs in CD3+ cells of LCa patients						
miRNA	Median healthy donor	Median LCa patients	Log difference	*T* test	Ratio LCa/healthy	fold change
hsa-miR-34a	3,25	4,78	−1,53	0,00	1,47	2,88
hsa-miR-21*	4,03	4,83	−0,80	0,00	1,20	1,74
hsa-miR-34b*	2,04	2,67	−0,63	0,01	1,31	1,55
hsa-miR-92a-1*	1,00	1,55	−0,54	0,00	1,54	1,46
hsa-miR-21	11,35	11,74	−0,39	0,01	1,03	1,31
hsa-miR-3668	0,42	0,79	−0,38	0,02	1,91	1,30
hsa-miR-1266	0,17	0,49	−0,32	0,02	2,82	1,25
hsa-miR-939	5,27	5,56	−0,29	0,04	1,05	1,22
hsa-miR-1273c	0,85	1,11	−0,26	0,04	1,31	1,20
hsa-miR-3123	0,37	0,63	−0,25	0,04	1,68	1,19
hsa-miR-3659	2,11	2,35	−0,24	0,02	1,11	1,18
hsa-miR-525-3p	1,62	1,80	−0,19	0,03	1,12	1,14
hsa-miR-513a-3p	1,24	1,43	−0,19	0,00	1,15	1,14

We reanalyzed the miRNA expression in CD3+ cells of LCa patients vs. healthy donors concerning the fold change of Leidinger et al.

### Validation of PKC family members as miR-34a targets by dual luciferase assay

**Figure 2:**
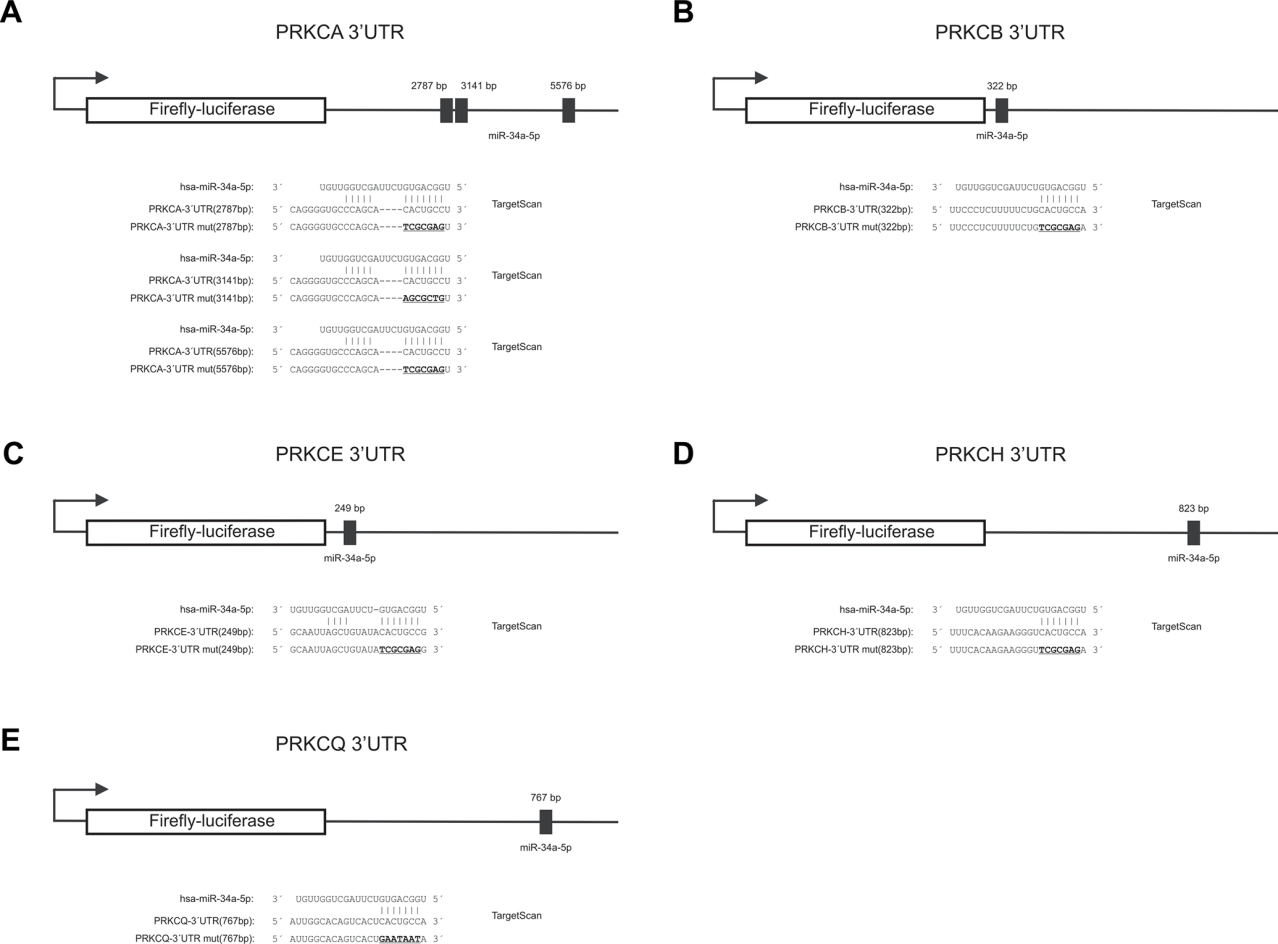
Schematic diagram of the reporter gene vectors including miR-34a-5p binding sites The location of the predicted binding sites of miR-34a-5p in the 3′UTRs of *PRKCA*, *PRKCB*, *PRKCE*, *PRKCH*, *PRKCQ* and additionally the sequences of the binding sites of miR-34a-5p as well as the mutated binding sites (underlined capital letters) are shown. (**A**)*PRKCA*-3′UTR, (**B**) *PRKCB*-3′UTR, (**C**) *PRKCE*-3′UTR, (**D**) *PRKCH*-3′UTR and (**E**) *PRKCQ*-3′UTR.

**Figure 3:**
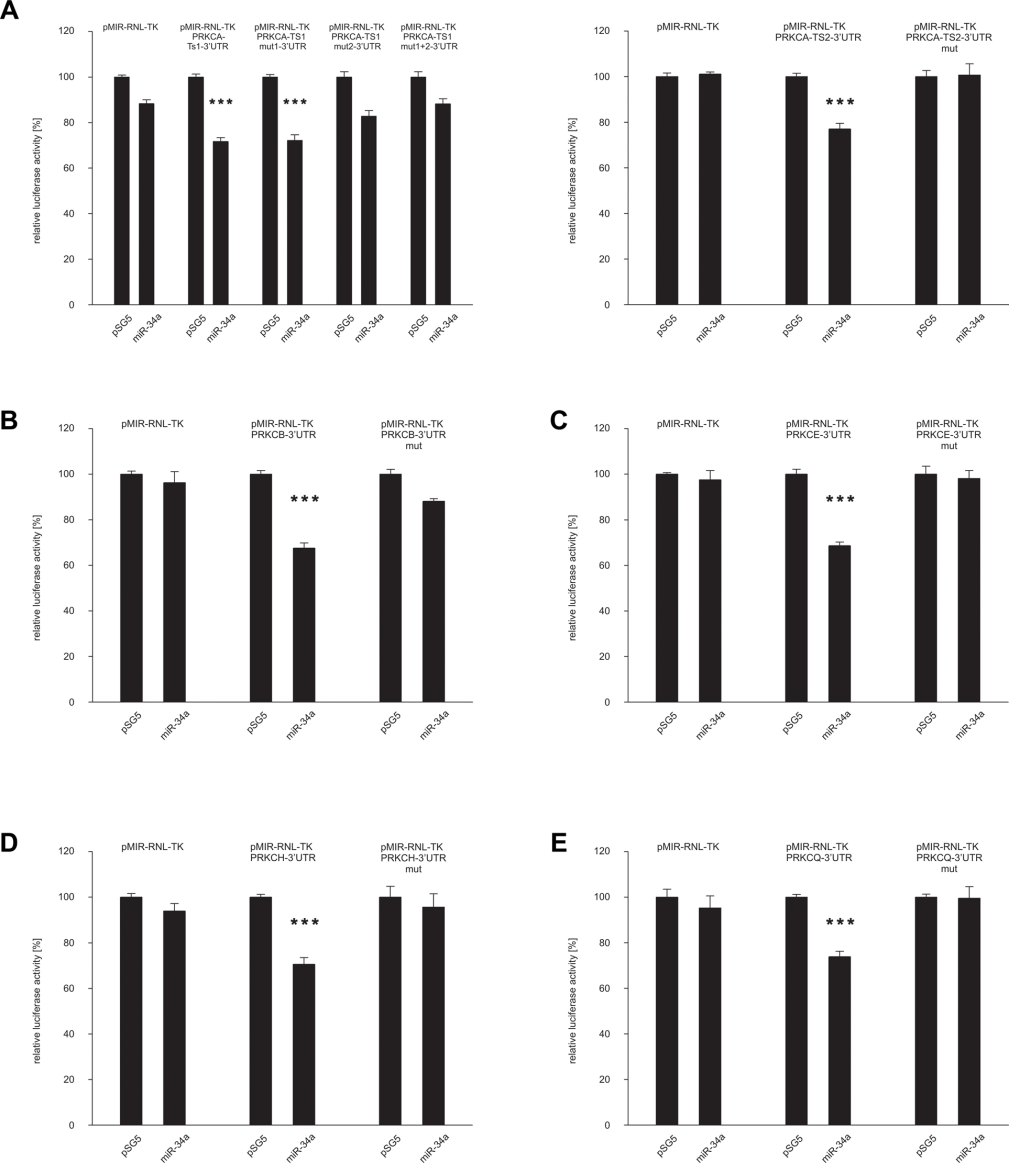
Dual luciferase reporter gene assays of the 3′UTRs of *PRKCA*, *PRKCB, PRKCE, PRKCH and PRKCQ*. HEK 293T cells were transfected with empty vectors, reporter gene constructs and miRNA-expression plasmids in the indicated combinations. The luciferase activity of the control vector experiment was set to 100%. The results represent the mean of at least three independent experiments carried out in duplicates. Three asterisks correspond to a *P* value lower than 0.001. Data are represented as mean +/− SEM. (**A**) *PRKCA*-3′UTR, (**B**) *PRKCB*-3′UTR, (**C**) *PRKCE*-3′UTR, (**D**) *PRKCH*-3′UTR and (**E**) *PRKCQ*-3′UTR.

We first identified a predicted target sequence at nucleotide 767 within the 3′UTR of *PRKCQ* (protein kinase C Q) as shown in Figure 2E. To experimentally confirm *PRKCQ* as target of miR-34a, we utilized the dual luciferase assay. We amplified the nucleotides 43–902 of the 3′UTR of *PRKCQ* via PCR and inserted the PCR product into the pMIR-RNL-TK reporter plasmid. In the following, the recombinant sequence is referred to as pMIR-RNL-TK-PRKCQ-3′UTR and the control reporter vector without insert as pMIR-RNL-TK. HEK 293T cells were cultivated and transfected both with reporter constructs and miR-34a expression plasmid. As shown in Figure 3E the luciferase activity of the pMIR-RNL-TK-PRKCQ-3′UTR reporter plasmid compared to pMIR-RNL-TK control vector was significantly reduced to 74% (*P* value 0.000002) by overexpression of miR-34a, indicating effective binding of miR-34a-5p to the 3′UTR of *PRKCQ*. As control the miR-34a binding site within the 3′UTR of *PRKCQ* was disrupted as indicated in Figure 2E. HEK 293T cells, which were transfected with the according recombinant plasmid referred to as pMIR-RNL-TK-PRKCQ-3′UTR mut showed a luciferase activity that was comparable to the activity found for cells transfected with the empty control vector. All dual luciferase assays were performed in duplicates and have been repeated at least 3 times.

The identification of *PRKCQ* as target of miR-34a-5p prompted us to test further potential targets, which also belong to the protein kinase C family. Therefore we analyzed all protein kinase C family members which contain a miR-34a-5p binding site in their 3′UTR, including *PRKCA, PRKCB, PRKCE* and *PRKCH*. As for *PRKCA*, we identified three potential target sequences for miR-34a-5p at nucleotides 2787, 3141 and 5576 within the 3′UTR (Figure 2A). For *PRKCB*-, *PRKCE*- and *PRKCH*-3′UTRs single potential target sites were predicted at nucleotide 322, 249 and 823 respectively as shown in Figure 2B–2D. As described above for *PRKCQ*, we generated PCR-amplifications spanning the potential binding sites, cloned the sequences into the pMIR-RNL-TK reporter vector and tested these reporter constructs in dual luciferase reporter assays. The pMIR-RNL-TK-PRKCA TS1, containing the first two binding sites and the pMIR-RNL-TK-PRKCA TS2 reporter constructs were coexpressed with the miR-34a expression vector in HEK 293T cells, leading to a significant decrease of the luciferase activity to 72% (*P* value 0.0000001) and to 77% (*P* value 0.00000007), respectively (Figure 3A). The luciferase activity of the pMIR-RNL-TK-PRKCA TS2 mut1 reporter vector with the disrupted first binding site of miR-34a was also decreased by miR-34a to 72% (*P* value0.000001) while pMIR-RNL-TK-PRKCA TS2 mut2 reporter showed no effect. For this reason only the second miR-34a binding site is responsive. The cotransfection of miR-34a with the pMIR-RNL-TL-PRKCB reporter plasmid showed a reduction of the luciferase activity to 68% (*P* value 0.0000000008) (Figure 3B). As shown in Figure 3C and 3D the luciferase activities of the pMIR-RNL-TL-PRKCE- and pMIR-RNL-TL-PRKCH reporter constructs were reduced by miR-34a overexpression to 68% (*P* value 0.0000004) and 70% (*P* value 0.0000003), respectively. In all cases the luciferase activities of the mutated reporter constructs were comparable to the activities found for cells transfected with the empty control vector (Figure 3A–3E). As above, all experiments have been repeated at least 3 times in duplicates.

### Effect of ectopic expression of miR-34a on PKC isozyme protein expression

**Figure 4:**
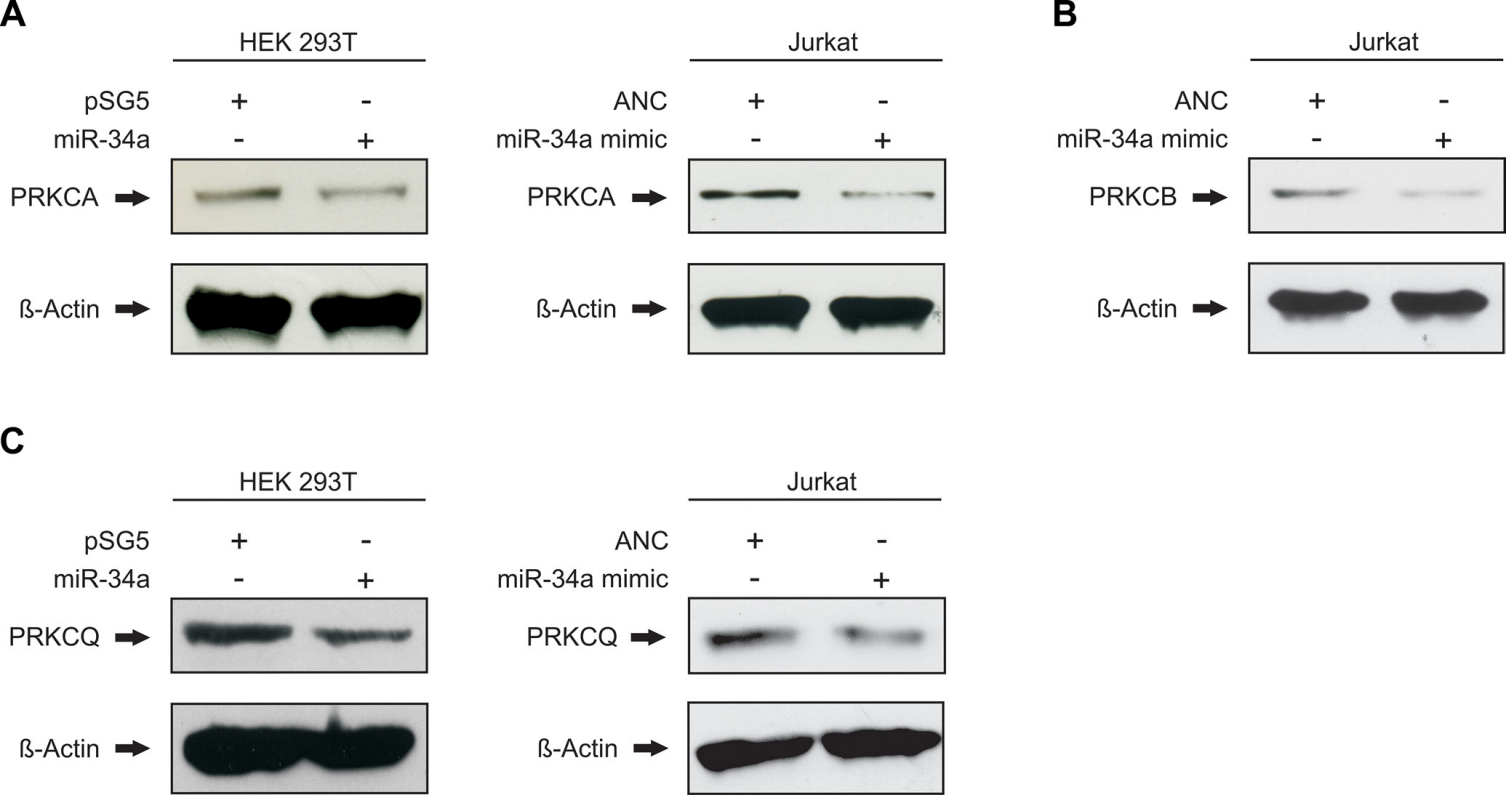
miR-34a-5p regulates the endogenous protein levels of PRKCA, PRKCB and PRKCQ HEK 293T were transfected either with empty control vector or miR-34a expression plasmid. Jurkat cells were transfected with nontargeting control (allstars negative control) or miR-34a-5p mimic. 48 h after transfection the endogenous protein levels of PRKCA (**A**), PRKCB (**B**) and PRKCQ (**C**) were detected by Western blotting using specific antibodies against PRKCA, PRKCB and PRKCQ. Beta-actin served as loading control.

**Figure 5:**
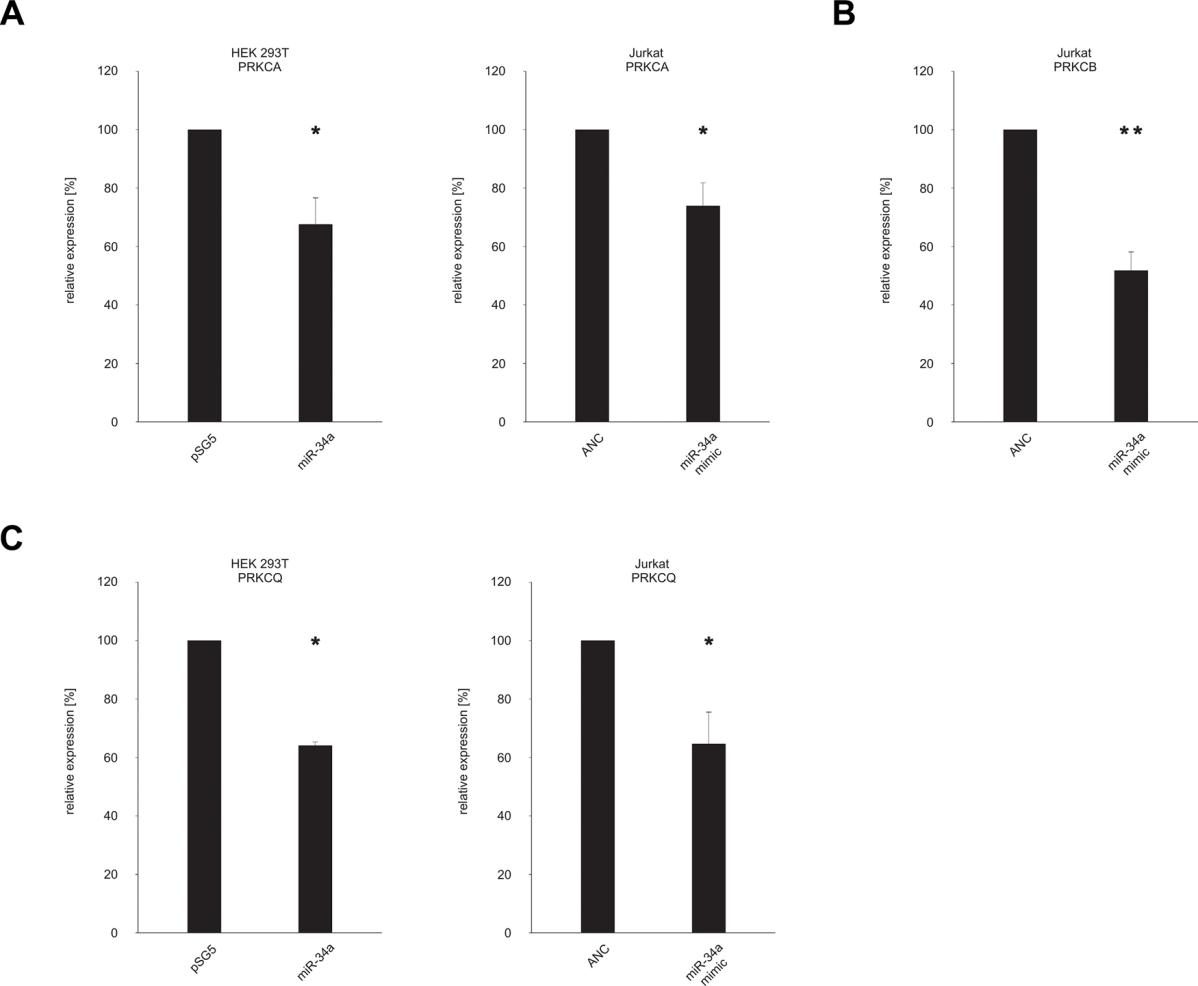
Quantification of the endogenous protein levels of PRKCA, PRKCB, PRKCH and PRKCQ The Western Blots shown in Figure 4A–4C were quantified by densitometry. The protein expression of PRKCA (**A**), PRKCB (**B**) and PRKCQ (**C**) was normalized according to the beta-actin signals of the appropriate samples. The results show the mean of three independent experiments. One asterisk correspond to a *P* value lower than 0.05 and two asterisks correspond to a *P* value lower than 0.01. Data are represented as mean +/− SEM.

With the binding of miR-34a-5p confirmed for all seven tested target sequences of the five protein kinase C family members, we next analyzed the downstream effect on the corresponding endogenous proteins. To this end, HEK 293T cells were again transfected either with the miR-34a expression vector pSG5-miR-34a or the empty control vector pSG5. Following transfection the overexpression of miR-34a-5p was confirmed by Northern blotting and qRT-PCR as shown in Supplementary Figure S1. The subsequent Western blot analysis was performed with specific antibodies against PRKCA, PRKCB and PRKCQ. The effect of miR-34a-5p overexpression on the endogenous protein level of PRKCE was not further tested, because Zhao and colleagues already showed the regulation of PRKCE by miR-34a-5p in glioma endothelial cells [29]. For each Western blot we compared HEK 293T cells that were transfected with pSG5-miR-34a to cells transfected with the empty pSG5 vector. We found reduced levels of PRKCA and PRKCQ in the cells transfected with miR-34a providing evidence for a biological effective binding of miR-34a to their corresponding target sequences of the protein kinase C family members (Figure 4A and 4C). Due to the low amount of PRKCB in HEK 293T cells (Supplementary Figure S2) only PRKCA and PRKCQ were analyzed in this cell line. Figure 4 shows one representative Western Blot out of 3 independent experiments. To further substantiate this conclusion and to test these effects in T-cells we repeated the Western blot experiments with an immortalized cell line of human T lymphocyte cells (Jurkat cells). The Comparison of Jurkat cells and HEK 293T cells showed high endogenous expression of PRKCA, PRKCB, and PRKCQ in this cell line (Supplementary Figures S2 and S3). The Jurkat cells were transfected with “allstars negative control” as a nontargeting control or with a miR-34a-5p mimic, which imitate the mature endogenous miR-34a-5p. Using the same specific antibodies as for the HEK 293T cells, we detected reduced amounts of PRKCA, PRKCB and PRKCQ in the cells transfected with miR-34a-5p mimic as compared to the cells that were transfected with the nontargeting control (Figure 4). Figure 4 shows one representative Western Blot out of 3 independent experiments. A quantification of the altered protein levels of PRKCA, PRKCB, and PRKCQ upon transfection of Jurkat and HEK 293T cells with the miR-34a-5p mimic is given in Figure 5. In the HEK 293T cells ectopic overexpression of miR-34a-5p reduced the PRKCA protein level to 68% (*P* value 0.02) and the PRKCQ protein level to 64% (*P* value 0.00001). In miR-34a-5p mimic transfected Jurkat cells the protein levels of the PKC isozymes decreased to 73% (*P* value 0.01) for PRKCA, to 52% (*P* value 0.001) for PRKCB and to 64% (*P* value 0.03) for PRKCQ. In summary we confirmed binding of miR-34-5p to the predicted target sequences of the five protein kinase C family members and demonstrated reduced protein expression of PRKCA and PRKCQ in HEK 293T cells and of PRKCA, PRKCB and PRKCQ in Jurkat cells.

## DISCUSSION

To understand the interference of miRNAs with biological pathways and disease mechanisms, it is essential to know their specific targets. While there is common agreement upon the idea of a single miRNA binding to a multitude of different target genes, the question of a possible common denominator of these targets remains largely unaddressed. Here, we identified binding of miR-34a-5p to the predicted target sequences of the five protein kinase C family members *PRKCA*, *PRKCB*, *PRKCE*, *PRKCH* and *PRKCQ* associated with reduced protein expression of PKC isozymes *PRKCA*, *PRKCB* and *PRKCQ* upon transfection with miR-34a-5p. There are as of now only few studies that demonstrate binding of a specific miRNA to members of a protein family. Recently, we reported binding of miR-145 to the proto-oncogene *ERG* (the v-ets avian erythroblastosis virus e26 oncogene homolog), which belongs to the large family of ETS (E26 transformation-specific) transcription factors [30]. The same miRNA also binds to *FLI-1*(Friend leukemia virus integration 1) and *ETS-1* (v-ets erythroblastosis virus E26 oncogene homolog 1), which are both members of the ETS family of transcription factors [31, 32]. We suggested that miR-145 might regulate different members of this transcription factor family towards suppression of cell growth [30]. With 29 genes belonging to the ETS family in humans, the hypothesis was, however, based only on circumstantial evidence. Our data on miR-34a-5p further substantiates the claim that single miRNAs simultaneously target different members of the same protein family, in our case isozymes of PKCs, to efficiently mediate cellular downstream effects.

As for the expression of miR-34a-5p in different biological specimens, we implemented a tissue miRNA atlas that includes the expression data of human miRNAs across 30 human tissues (https://ccb-web.cs.uni-saarland.de/tissueatlas/) [33]. Most recently, we added whole blood, blood cells, serum, plasma, urine and saliva. The tissue distribution for miR-34a-5p across the solid tissues and the body fluids is given in Supplementary Figures 4 and 5. As for the evolutionary conservation, we found 100% sequence identity between the mature miRNA of homo sapiens and 22 other species. The list of miRBase identifiers includes hsa-miR-34a-5p, mmu-miR-34a-5p, rno-miR-34a-5p, dre-miR-34a, ggo-miR-34a, age-miR-34a, ppa-miR-34a, ppy-miR-34a, ptr-miR-34a, mml-miR-34a-5p, sla-miR-34a, lla-miR-34a, mne-miR-34a, bta-miR-34a, cfa-miR-34a, ssc-miR-34a, eca-miR-34a, tgu-miR-34a, cgr-miR-34a, ccr-miR-34, ipu-miR-34a, chi-miR-34a. Seven species showed also an identical sequence but with an uracil added to the mature form of the miRNAs. The identifiers are chi-miR-34a, gga-miR-34a-5p, xtr-miR-34a, mdo-miR-34a-5p, tch-miR-34a-5p, tch-miR-34a-5p, oha-miR-34a-5p. As for variants of miR-34a, the database MiRNASNP shows rs201359809, rs35301225, and rs72631823, the latter of which has been associated with increased levels of miR-34a in pancreatic beta cells ([34]).

MiR-34a plays a pivotal role in the carcinogenesis of multiple cancer subtypes. As a tumor suppressor, miR-34a is down-regulated for example in glioblastoma, meningioma, lung carcinoma, prostate carcinoma and colon cancer, targeting Delta-like protein1 (DLL1), jun proto-oncogene (JUN), MET proto-oncogene (MET), CD44 molecule (CD44) and Notch-1 [35–39]. However, in serum or whole blood of cancer patients miR-34a is found upregulated [40–43]. Likewise, PKC isozymes are strongly associated with human cancers. Specifically, PRKCA overexpression is associated with increased proliferation and decreased apoptosis in gliomas, bladder cancer and prostate carcinomas [44–46]. Aberrant expression of PRKCB is also linked to glioblastoma, breast and prostate cancer [16, 47, 48] as well as PRKCE overexpression [18, 49, 50]. In addition, PRKCH is involved in drug resistance and regulation of apoptosis in breast cancer and PRKCQ promotes epithelial to mesenchymal transition (EMT) in breast cancer stem cells [19, 51]. Even though the PKC isozymes were aberrantly expressed in a variety of cancers, the correlation between these expression changes and tumorigenesis are still not completely elucidated [21]. With the identification of the PKC isozymes *PRKCA*, *PRKCB*, *PRKCE*, *PRKCH* and *PRKCQ* as targets of miR-34a-5p, we contribute to understand the mechanisms of regulation of PKC isozymes and substantiate the potential of miR-34a as pharmacological target for cancer treatment as shown in various preclinical studies [52–55] and a clinical trial (NCT01829971).

Besides its central role in cancer, miR-34a is also crucial in other diseases and it is furthermore fundamental in physiological processes like apoptosis, cell cycle arrest, proliferation, senescence and differentiation [56–59]. As indicated above we identified a highly increased abundance of hsa-miR-34a in CD3+ T-cells of lung cancer patients as compared to healthy individuals. Notably, the targeted PKC isozymes are involved in cell signaling trough the immunological synapse (IS) in both the adaptive and the innate immune system [26]. In detail, all PKC isozymes, which were analyzed in our study, are expressed by immune cells and exhibit important functions in immune cell activation. PRKCQ, PRKCE and PRKCH regulate gene transcription downstream of the T-cell receptor (TCR) and accumulate at the IS [60–62]. Additionally a study of Grybko et al. showed that PRKCA translocates in synergy with PRKCQ to the IS of cytotoxic T-cells to mediate lytic granule exocytosis [63]. PRKCB coordinates together with PRKCD the responsiveness of migratory human blood T-lymphocytes [64]. Various PKC knockout studies analyzing *PRKCA*-, *PRKCB*-, *PRKCE*-, *PRKCH*- or *PRKCQ*-deficient mice observed defects in immune cell activation and function [65–69]. It is legitimate to hypothesize that the overexpression of miR-34a impacts the activation and functionality of immune cells via decreased levels of PRKCA, PRKCB, PRKCE, PRKCH or PRKCQ.

In summary, our network analysis identified miR-34a as one of the miRNAs with the most interactions with human diseases. Among the analyzed human blood cells, CD3+ cells showed the strongest deregulation of miR-34a in patients with lung cancer. Computational analysis identified potential binding sites in isozymes of the protein kinase C. We experimentally confirmed binding of miR-34a-5p to target sequences within the 3′UTRs of five PRKCQ family members. As a result of miRNA overexpression we found reduced endogenous protein levels of PKC isozymes. This study shows that one of the most important disease associated miRNAs target several members of the PKC protein family.

## MATERIALS AND METHODS

### Cell lines, tissue culture

The human HEK 293T and Jurkat cell lines were purchased from the German collection of microorganisms and cell cultures (DSMZ). The Authentication of these cell lines was ensured using STR DNA typing by the DSMZ. HEK 293T cells were grown in DMEM (Life Technologies GmbH, Darmstadt, Germany) supplemented with 10% Fetal bovine serum (Biochrom GmbH, Berlin, Germany), Penicillin (100 U/mL), Streptomycin (100 μg/mL). Cells were passaged for less than 6 months after receipt.

Jurkat cells were grown in RPMI1640 (Life Technologies GmbH, Darmstadt, Germany) supplemented with 10% Fetal bovine serum (Biochrom GmbH, Berlin, Germany), Penicillin (100 U/mL), Streptomycin (100 μg/mL). Cells were passaged for less than 6 months after receipt.

### Dual luciferase reporter assays

Usually 10^5^ HEK 293T cells per well were seeded in a 24-well format. The next day the cells were transfected with 0.2 μg/well reporter construct and 0.8 μg/well miRNA expression plasmid using PolyFect (Qiagen, Hilden, Germany) according to the manufacturer's protocol. Luciferase reporter assays were performed 48 hours post transfection corresponding to Dual-Luciferase^®^ Reporter Assay System Protocol (Promega, Mannheim, Germany).

### Western blot

For Western Blot analysis of PKCs 2 × 10^5^ HEK 293T cells per well were seeded in 6-well plates. After 24 hours they were transfected using PolyFect transfection reagent (Qiagen, Hilden, Germany) either with 2 μg of pSG5 empty vector or with 2 μg of miR-34a expression plasmid following the manufacturer's instructions. Jurkat cells were seeded out in a density of 2.5 × 10^5^ cells per well of a 6-well plate and immediately transfected either with the appropriate negative control or with hsa-miR-34a-5p miScript miRNA Mimic (MIMAT0000255: 5′UGGCAGUGUCUUAGCUGGUUGU) using HiPerFect transfection reagent (Qiagen, Hilden, Germany) according to the manufacturer's protocol.

48 hours post transfection the cells were lysed using 2× lysis buffer (130 mM Tris/HCl, 6% SDS, 10% 3-Mercapto-1,2-propandiol, 10% glycerol). 30 μg of whole cell lysate were separated in a 10% SDS gel and electroblotted on a nitrocellulose membrane (Whatman, GE Healthcare, Freiburg, Germany). The primary antibodies used were anti-PRKCA polyclonal rabbit antibody (2056S, Cell Signaling Technology, Danvers, United States), anti-PRKCB monoclonal mouse antibody (ABIN967769, antibodies-online GmbH, Aachen, Germany), anti-PRKCQ monoclonal rabbit antibody (E1I7Y, Cell Signaling Technology, Danvers, United States) and anti-β-actin monoclonal mouse antibody (AC-15, Sigma Aldrich, Munich, Germany). The corresponding secondary antibodies were purchased from Sigma Aldrich (Sigma Aldrich, Munich, Germany).

### Plasmids

The pSG5-miR-34a expression vector was generated by Eurofins Genomics containing the nucleotides 9151617-9151816 of chromosome 1 (Eurofins Genomics, Ebersberg, Germany). All 3′UTRs were cloned into the pMIR-RNL-TK vector, which was described in Beitzinger et al. using the SpeI, SacI restriction sites [70]. The nucleotides 2578-3398 and 5307-6637 of the *PRKCA* 3′UTR (NM_002737.2), nucleotides 4-737 of the *PRKCB* 3′UTR (NM_002738.6), nucleotides 44-846 of the *PRKCE* 3′UTR (NM_005400.2), nucleotides 86-868 of the *PRKCH* 3′UTR (NM_006255.4) and nucleotides 43-902 of the *PRKCQ* 3′UTR (NM_006257.4) were amplified via PCR using specific primers (Supplementary Table S1) from testis cDNA. All predicted hsa-miR-34a-5p target sites were mutated by site-directed mutagenesis with the QuickChange II Site-Directed Mutagenesis Kit (Agilent Technologies, Santa Clara, United States) using specific primers (Supplementary Table S1).

### Northern blot

For total RNA extraction using Qiazol (Qiagen, Hilden, Germany) 2 × 10^5^ HEK 293T cells were seeded out in each well of a 6-well plate. 24 hours later cells were transfected either with 2 μg of pSG5 empty vector or with 2 μg of miR-34a expression plasmid according to the PolyFect transfection protocol. 48 hours after transfection the total RNA was isolated, separated by 12% denaturing urea-polyacrylamide gel and transferred to a nylon membrane Hybond N (Amersham, GE Healthcare, Freiburg, Germany) by semi-dry electroblotting. After chemical crosslinking of the RNA the membrane was incubated overnight with the specific radiolabeled probe against hsa-miR-34a-5p (TGGCAGTGTCTTAGCTGGTTGTCCTGTCTC). The construction of the hsa-miR-34a probe was carried out according to the manual of the miRVana probe construction kit (Life Technologies GmbH, Darmstadt, Germany). The next day the membrane was washed twice for 15 min with 5× SSC and 1% SDS and twice for 15 min with 1× SSC and 1% SDS and then exposed for 24 hours to a storage phosphor screen.

### Quantitative real time PCR (qRT-PCR)

The expression of hsa-miR-34a-5p, *PRKCA, PRKCB, PRKCE, PRKCH* and *PRKCQ* was analyzed using qRT-PCR with the StepOnePlus Real-Time PCR System (Applied Biosystems, Foster City, United States) and the miScript PCR System (Qiagen, Hilden, Germany) or the QuantiTect Primer Assay respectively (Qiagen, Hilden, Germany) corresponding to the manufacturer's manual. In brief, 200 ng total RNA was reverse transcribed into cDNA using the miScript RT II Kit with the miScript HiFlex Buffer (Qiagen, Hilden, Germany). RNU48 or -β-actin served as endogenous control served for miRNA and mRNA respectively.

### Data analysis

Statistical analysis of the luciferase assays and the Western Blots was carried out with SigmaPlot 10 (Systat, Chicago, United States) applying student's *t*-test. Quantification of the Western Blots was performed with Quantity One analysis software (Bio-Rad Laboratories GmbH, München, Germany)

## SUPPLEMENTARY MATERIALS FIGURES AND TABLES


