# Supplementary material for: Identification of miR-34a-target interactions by a combined network based and experimental approach
Source: Oncotarget. 2016 Apr 29;7(23):34288–99. doi: 10.18632/oncotarget.9103 (PMC5085156; doi:10.18632/oncotarget.9103)
Supplement: Supplementary file 1 [file oncotarget-07-34288-s001.pdf]

Identification of miR-34a-target interactions by a combined network based and experimental approach

Supplementary Materials

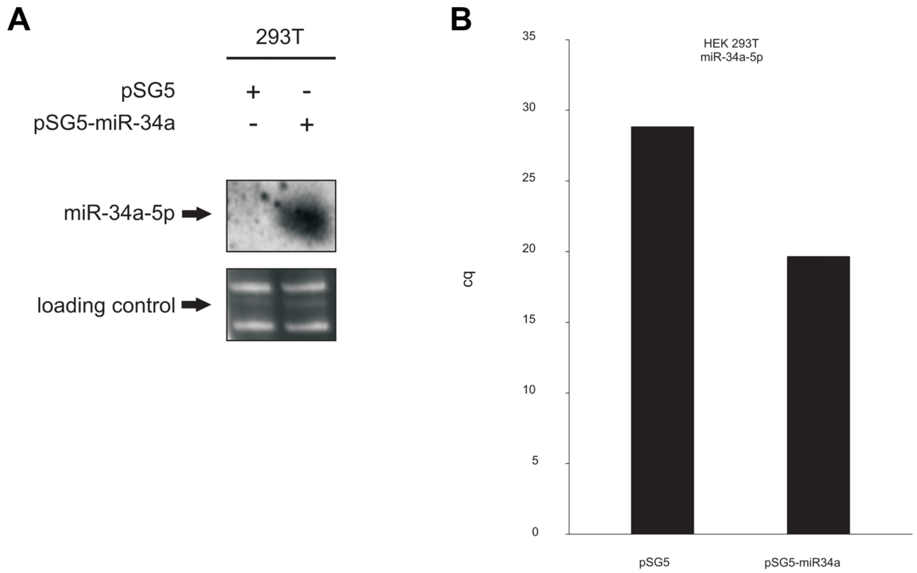

Supplementary Figure S1: Analysis of miR-34a-5p expression by Northern blotting and qRT-PCR. HEK 293T cells were transfected either with the empty pSG5 vector or the miR-34a expression plasmid. 48 h post transfection the total RNA was isolated and analyzed either by Northern blotting using a specific radioactive labeled probe against miR-34a-5p (**A**) or by qRT-PCR using a specific hsa-miR-34a-5p primer (**B**).

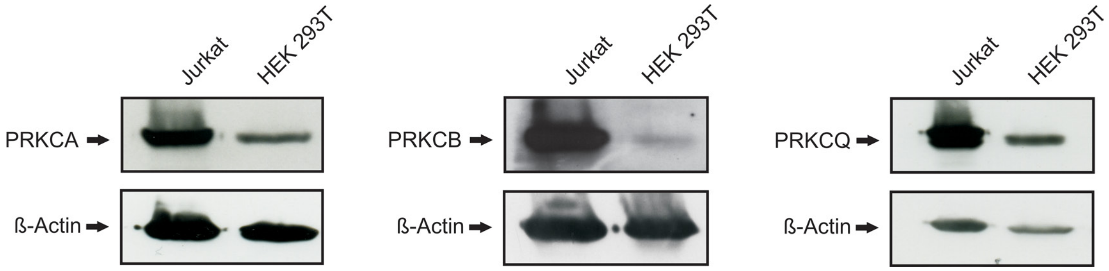

Supplementary Figure S2: Western Blot analysis of the endogenous PKC isozymes in Jurkat and HEK 293T cells. The endogenous protein levels of PRKCA, PRKCB and PRKCQ in both cell lines were detected by Western blotting using specific antibodies against PRKCA, PRKCB and PRKCQ. Beta-actin served as loading control.

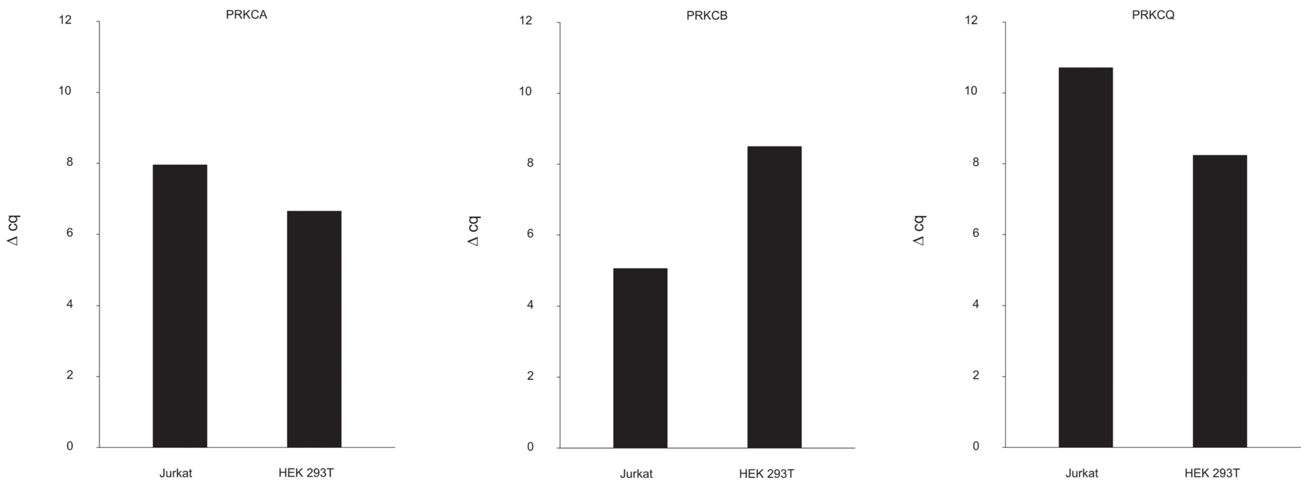

Supplementary Figure S3: qRT-PCR analysis of the PKC isozymes mRNA in Jurkat and HEK293T cells. The endogenous mRNA levels of *PRKCA*, *PRKCB* and *PRKCQ* in both cell lines were detected by qRT-PCR using specific primers for *PRKCA*, *PRKCB* and *PRKCQ*.

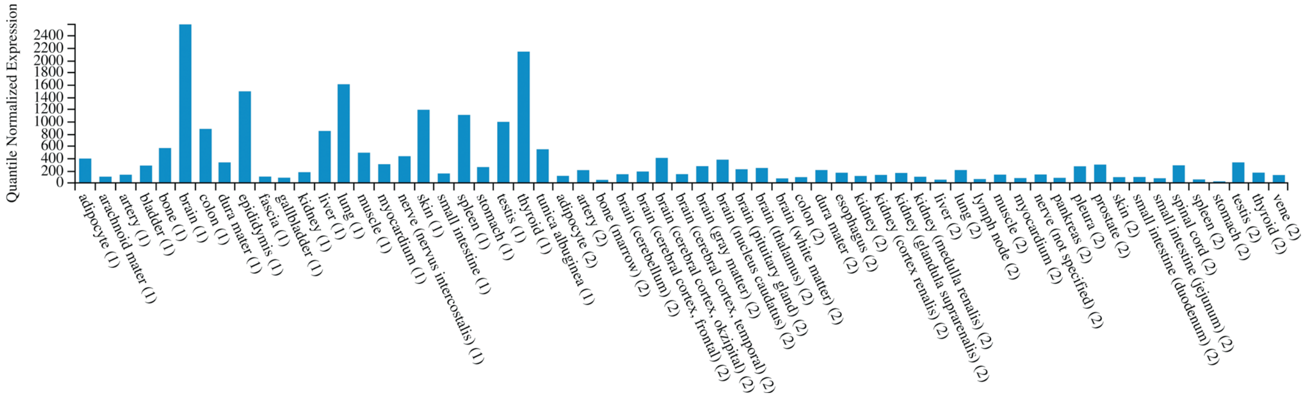

Supplementary Figure S4: Expression of miR-34a in different tissues.

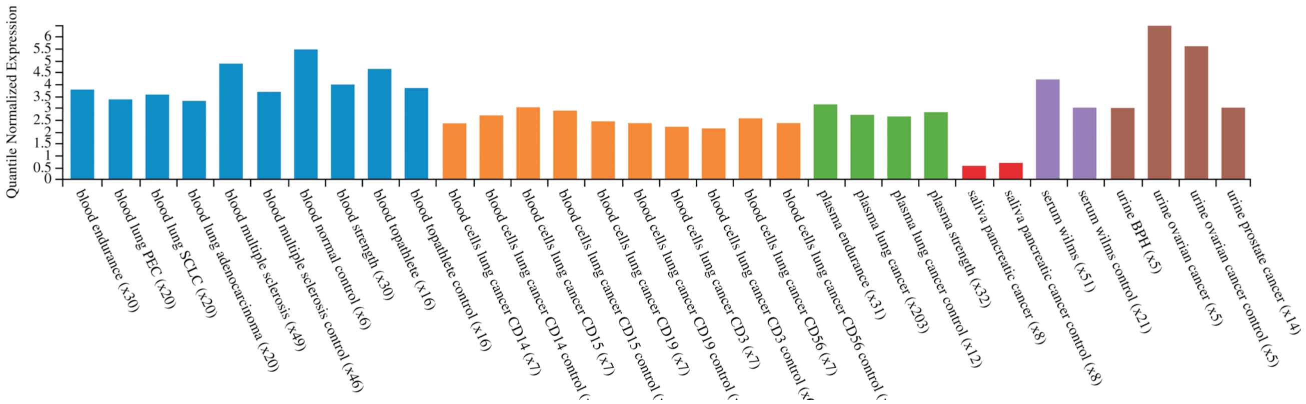

Supplementary Figure S5: Expression of miR-34a in different body fluids.

Supplementary Table S1: Sequences of cloning and mutagenesis primers

**cloning primers**	*(restriction sites are underlined)*
**name**	**sequence**
5′-PRKCA TS1-SpeI	ggactagtgaggaacacatgaggttagg
3′-PRKCA TS1-SacI	cgagctccactccagtgacttcctttc
5′-PRKCA TS2-SpeI	ggactagtcatccaacagccttacagtg
3′-PRKCA TS2-SacI	cgagctccatgcaaacggagattagag
5′-PRKCB-SpeI	ggactagtgatgtgtagatctccgtccttc
3′-PRKCB-SacI	cgagctcgctagagtgactgtgttgtttc
5′-PRKCE-SpeI	ggactagtgcagagaagactcctgtgttg
3′-PRKCE-SacI	cgagctccagctctcatgtgtcactcg
5′-PRKCH-SpeI	ggactagtcagccttagaacaagaaccttacc
3′-PRKCH-SacI	cgagctcctggtcactgacatgagtg
5′-PRKCQ-SpeI	ggactagtggaactggttcaagagacactg
3′-PRKCQ-SacI	cgagctcgatcttgaatgatgcctacgg

**mutagenesis primers**	*(mutated sites are shown in capital letters)*
**name**	**sequence**
5′-PRKCA TS1-BS1mut	caggggtgcccagcaTCGCGAGtgagggacaacagac
3′-PRKCA TS1-BS1mut	gtctgttgtccctcaCTCGCGAtgctgggcacccctg
5′-PRKCA TS1-BS2mut	gtccttgcccctgagAGCGCTGtgattgccagggcc
3′-PRKCA TS1-BS2mut	ggccctggcaatcaCAGCGCTctcaggggcaaggac
5′-PRKCA TS2-mut	gcacccccgacctgatctccTCGCGAGagattttcccatgctcctagg
3′-PRKCA TS2-mut	cctaggagcatgggaaaatctCTCGCGAggagatcaggtcgggggtgc
5′-PRKCB-mut	gctttctttccctctttttctgTCGCGAGatattcacccccaaccatcc
3′-PRKCB-mut	ggatggttgggggtgaatatCTCGCGAcagaaaaagagggaaagaaagc
5′-PRKCE-mut	gcaattagctgtataTCGCGAGgtgtttggaccattg
3′-PRKCE-mut	caatggtccaaacacCTCGCGAtatacagctaattgc
5′-PRKCH-mut	ctctttttcacaagaagggtTCGCGAGacaacagcacagtcagc
3′-PRKCH-mut	gctgactgtgctgttgtCTCGCGAacccttcttgtgaaaaagag
5′-PRKCQ-mut	gtattggcacagtcactGAATAATattctcacactataac
3′-PRKCQ-mut	gttatagtgtgagaatATTATTCagtgactgtgccaatac
